# Distinct stimulus-dependent neutrophil dynamics revealed by real-time imaging of intestinal mucosa after acute injury

**DOI:** 10.1093/pnasnexus/pgac249

**Published:** 2022-11-04

**Authors:** Veronica Azcutia, Matthias Kelm, Seonyoung Kim, Anny-Claude Luissint, Sven Flemming, Lisa Abernathy-Close, Vincent B Young, Asma Nusrat, Mark J Miller, Charles A Parkos

**Affiliations:** Department of Pathology, University of Michigan; Ann Arbor, MI 48109, USA; Department of Pathology, University of Michigan; Ann Arbor, MI 48109, USA; Department of Internal Medicine, Washington University School of Medicine; Saint Louis, MO 63110, USA; Department of Pathology, University of Michigan; Ann Arbor, MI 48109, USA; Department of Pathology, University of Michigan; Ann Arbor, MI 48109, USA; Department of Internal Medicine/Division of Infectious Diseases, University of Michigan; Ann Arbor, MI 48109, USA; Department of Internal Medicine/Division of Infectious Diseases, University of Michigan; Ann Arbor, MI 48109, USA; Department of Microbiology and Immunology, University of Michigan; Ann Arbor, MI 48109, USA; Department of Pathology, University of Michigan; Ann Arbor, MI 48109, USA; Department of Internal Medicine, Washington University School of Medicine; Saint Louis, MO 63110, USA; Department of Pathology, University of Michigan; Ann Arbor, MI 48109, USA

**Keywords:** intestinal mucosa, inflammation, neutrophils, migration, intravital microscopy

## Abstract

Clinical symptoms in many inflammatory diseases of the intestine are directly related to neutrophil (PMN) migration across colonic mucosa and into the intestinal lumen, yet in-vivo studies detailing this process are lacking. Using real-time intravital microscopy and a new distal colon loop model, we report distinct PMN migratory dynamics in response to several models of acute colonic injury. PMNs exhibited rapid swarming responses after mechanically induced intestinal wounds. Similar numbers of PMNs infiltrated colonic mucosa after wounding in germ-free mice, suggesting microbiota-independent mechanisms. By contrast, acute mucosal injury secondary to either a treatment of mice with dextran sodium sulfate or an IL-10 receptor blockade model of colitis resulted in lamina propria infiltration with PMNs that were largely immotile. Biopsy wounding of colonic mucosa in DSS-treated mice did not result in enhanced PMN swarming however, intraluminal application of the neutrophil chemoattractant LTB_4_ under such conditions resulted in enhanced transepithelial migration of PMNs. Analyses of PMNs that had migrated into the colonic lumen revealed that the majority of PMNs were directly recruited from the circulation and not from the immotile pool in the mucosa. Decreased PMN motility parallels upregulation of the receptor CXCR4 and apoptosis. Similarly, increased expression of CXCR4 on human PMNs was observed in colonic biopsies from people with active ulcerative colitis. This new approach adds an important tool to investigate mechanisms regulating PMN migration across mucosa within the distal intestine and will provide new insights for developing future anti-inflammatory and pro-repair therapies.

Significance Statement It is well recognized that neutrophils (PMNs) represent a double-edged sword, playing a critical role in first-line immune defense while also contributing to tissue damage in many diseases. In the gut, dysregulated PMN infiltration into mucosal epithelia is a pathological hallmark of conditions such as ulcerative colitis that strongly correlates with clinical symptoms. Unfortunately, studies investigating migration of PMNs within intestinal mucosa in vivo and in real-time are limited. Here, we employed a novel distal colon loop and intravital microscopy in the mouse to visualize for first time distinct PMN extravascular migratory dynamics resulting from specific injury conditions that mirror different pathophysiological aspects of ulcerative colitis.

## Introduction

Polymorphonuclear neutrophils (PMNs) are the first immune responders to infection or injury and play a critical role in clearing invading pathogens and promoting restitution of tissue homeostasis (1). The important role of PMNs in mucosal repair is highlighted in studies demonstrating that depletion of PMNs leads to significantly impaired wound healing in vivo (2, 3). However, while necessary to resolve infection and promote tissue repair, excessive tissue infiltration by PMNs and associated by-stander tissue damage are hallmarks of a number of chronic pathologic conditions such as inflammatory bowel disease (IBD) (4, 5). In IBD, transepithelial migration of PMNs and their accumulation within intestinal crypts to form crypt abscesses parallels symptoms in patients with active ulcerative colitis (UC) (6, 7). Given the clinical consequences of dysregulated PMN transmigration in the gut, increased understanding of PMN trafficking in vivo is crucial for gaining new insights into strategies aimed at reducing excessive PMN infiltration of mucosal tissues during inflammatory disease.

While mechanisms regulating PMN migration out of the microcirculation are relatively well described (8, 9), subsequent events including PMN egress through the interstitial space and migration across polarized epithelial monolayers into the lumen of the gut remain poorly understood. In-vitro studies using transwell-based transmigration systems have identified key PMN and intestinal epithelial proteins that play a role in regulating transepithelial migration (TEpM) (6, 7). Such in-vitro approaches have yielded important mechanistic insights in terms of the multistep nature of PMN TEpM, yet these models fail to recapitulate the complex environmental cues that guide PMN behavior in vivo. Another limitation of in-vitro approaches is the short life span of PMNs and activation of PMNs that inevitably occurs during isolation from blood or bone marrow (10, 11). More recently, elegant surgical models have been developed to quantify stimulus specific PMN migration into the ileum and proximal colon in vivo in response to different PMN chemoattractants such as fMLF, CXCL1, and LTB_4_ (12–14). While these end point models of PMN TEpM in vivo have confirmed the importance of key mediators including ICAM-1, JAM-A, CD11b/CD18, and CD47 in regulating intestinal PMN trafficking (13–15), systems for real time imaging of PMN in the gut have yet to be explored due in part to the complex nature of the mucosal architecture and intestinal motility.

To better study extravascular migratory dynamics of PMNs following focal acute intestinal injury and during colitis, we optimized an approach employing intravital microscopy (IVM) in the distal colon. By tracking real-time single cell migration, we demonstrate that PMNs exhibit robust swarming responses following acute biopsy induced wounding of the colonic mucosa. In contrast, PMN motility and swarming were reduced in the distal colon during acute DSS-induced injury. Importantly, observed differences in motility correlated with the altered expression of key PMN surface receptors, including CXCR4 and CD11b, and with altered levels of apoptosis. Here, we detail a new approach for studying immune cell trafficking in vivo that will increase our understanding of PMN migratory dynamics during intestinal inflammation.

## Results

### A new model to study PMN migration into the distal colon in vivo

**Fig. 1.**
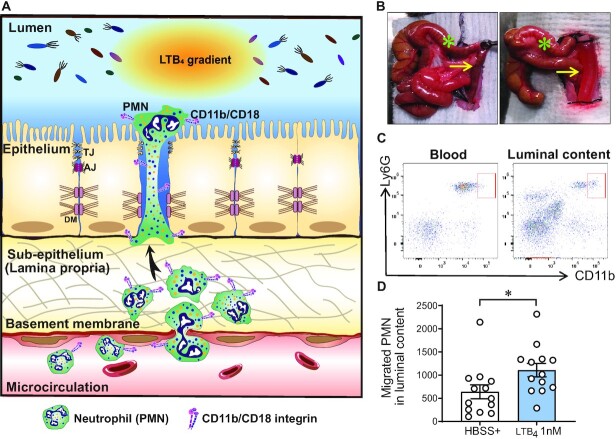
The DCL as a new model to study PMN recruitment. (A) Schematic overview of PMN migration from the circulation to the intestinal lumen in the DCL model of intestinal inflammation. Extravasation of PMNs from the circulation is stimulated by an inflammatory stimulus (i.e. TNFα and IFNγ stimulation or DSS-induced colitis). Subsequent PMN transepithelial migration (TEpM) is induced by intraluminal injection of PMN-specific chemoattractant (i.e. LTB_4_, CXCL1, or fMLF). (B) Images show proximal (green asterisk) and distal colon (yellow arrow), before (left image) and after (right image) intraluminal injection of a solution containing a PMN chemoattractant. (C) Representative flow cytometry plot showing the upregulation of CD11b on transmigrating PMNss (CD45^+^Ly6G^+^CD11b^high^) compared to PMN from peripheral blood (CD45^+^Ly6G^+^ CD11b^low^). (D) LTB_4_ (1 nM) significantly increases PMN TEpM in compared to control (HBSS^+^). Prior DCL, mice were injected i.p. with a cytokine cocktail containing IFNγ (100 ng) and TNFα (100 ng) 24 h. Graph represents quantification of migrated PMNs into luminal content by flow cytometry. Data are mean ± SEM of three independent experiments (*n* = 13 mice per group; *P < 0.05 as determined by Mann–Whitney *U* test).

As highlighted in Fig. 1A, much less is known about interactions that facilitate PMN trafficking across interstitium and TEpM into the intestinal lumen than the well characterized process of transendothelial migration. We developed a model to investigate PMN TEpM in the distal colon (termed DCL for distal colon loop), which accurately recapitulates the anatomic site associated with PMN influx and crypt abscesses formation in UC as well as in the DSS model of colitis in mice. In a fashion analogous to ileal (14–16) and proximal colon loop models (13, 15), we used an exteriorized segment of the distal colon, ensuring adequate blood supply (Fig. 1B). Noting that upregulation of CD11b occurs during PMN activation and transepithelial migration, we used CD11b surface expression levels to distinguish between PMNs that have undergone TEpM (CD45^+^CD11b^high^Ly6G^+^) from circulating PMNs (CD45^+^CD11b^low^Ly6G^+^) that might contaminate the luminal contents during surgery (Fig. 1C and Fig. S1A and B, Supplementary Material). Quantification by flow cytometry revealed that intraluminal injection of LTB_4_ resulted in a significant increase in PMN TEpM compared to control (Fig. 1D). Furthermore, CXCL1 (500 ng) also induced robust PMN TEpM in the DCL model (Fig. S1C, Supplementary material), as previously described in similar ileal loop model (14, 16), indicating that in-vivo TEpM is not limited to stimulation by LTB_4_. These findings demonstrate feasibility of accurate quantification of PMN TEpM in the distal colon in vivo.

### Reduced PMN motility in the colon during colitis

**Fig. 2.**
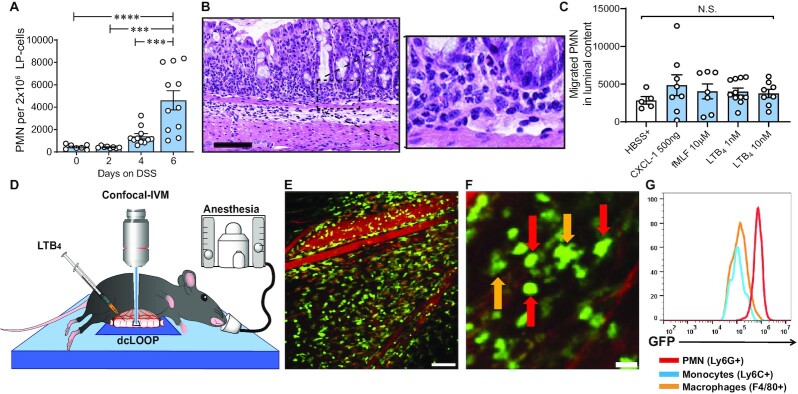
Visualizing PMN infiltration in the intestinal mucosa in a model of experimental colitis. (A) Quantification of PMNs infiltrating lamina propria upon 2.5% DSS. Graph represents number of PMNs per 2 × 10^6^ cells of lamina propria cells. Data are mean ± SEM of three independent experiments (*n* = 7 to 10 mice per group; ***P < 0.001; ^****^P < 0.0001 by one-way ANOVA). (B) Representative images of H&E staining histology showing PMNs infiltrating the distal colon lamina propria in response to DSS at day 4. Scale bar: 100 μm. (C) PMN TEpM in DSS-treated mice: DCL model was performed at day 4 of DSS-induced colitis and PMN TEpM was determined after 1 h of the intraluminal injection of different chemoattractants. Graph represents number of PMNs migrated into luminal content. Data are means ± SEM of two independent experiments (*n* = 5 to 10 mice per group; Not significant differences (N.S.) were found by one-way ANOVA). (D) Schematic model illustrating the IVM imaging set-up in the DCL model. (E) Representative confocal-IVM image of the subepithelial space of *LysM^eGFP/+^*mice treated with DSS for 4 days showed robust GFP^+^PMNs infiltrating the lamina propria. Scale bar: 200 μm. (F) Magnified confocal-IVM image of the subepithelial space in DSS treated *LysM^eGFP/+^*mice showed brighter GFP expression on PMN (red arrows) with rounded morphology compared to irregular shaped and GFP-dim macrophages (orange arrows). Scale bar: 50 μm. (G) GFP expression after gating on PMNs (CD45^+^CD11b^+^Ly6G^+^), monocytes (CD45^+^CD11b^+^Ly6G^−^Ly6C^high^F4/80^low^), and macrophages (CD45^+^CD11b^+^ Ly6G^−^Ly6C^low^F4/80^high^) as determined by flow cytometry.

Having demonstrated stimulus-dependent TEpM in the distal colon in vivo, we adapted the DCL model to investigate PMN trafficking dynamics during DSS-induced inflammation using IVM. Initial quantification of PMN numbers in the subepithelial space, or lamina propria (LP), revealed robust accumulation of PMNs 4 to 6 days after administration of DSS (Fig. 2A). We then performed surgical experiments to assess PMN TEpM in the DCL of mice that had been subjected to DSS for 4 days. Surprisingly, despite significant sub-epithelial accumulation of PMNs after 4 days of DSS (Fig. 2B), intraluminal injection of PMN chemoattractants (CXCL1, fMLF, LTB_4_) failed to induce increased PMN TEpM into distal colonic lumen relative to control (Fig. 2C). Analogous experiments after 6 days of DSS were performed but were not feasible due to extreme friability of the colonic mucosa and frequent rupture. We considered the possibility that a diffuse inflammatory microenvironment present during colitis might result in widespread PMN activation and arrest in the lamina propria, effectively desensitizing cells to further stimulation. To investigate this possibility, we administered DSS to *LysM^eGFP/+^*mice for 4 days followed by assessment of PMN migration in the lamina propria of distal colon by IVM (Fig. 2D and E). While it is well appreciated that lysozyme M is expressed on all myelomonocytic cells, expression is highest in PMNs relative to monocytes or macrophages (Fig. 2F and G). Thus, PMNs could be easily identified by assessing intensity of the LysM-driven GFP signal combined with characteristic PMN morphology as has been described previously (17–19). Consistent with flow cytometric quantification of luminal PMNs in Fig. 2C, very little migration of lamina propria PMNs was observed by IVM in response to intraluminal application of indicated chemoattractants (Fig. 2E, and Movie S1, Supplementary Material). PMNs had apparently become functionally unresponsive, and the chemoattractant gradient was not effective in overcoming other cues. We performed IVM at early time points after exposure to DSS in an attempt to visualize motile PMNs even though minimal PMN recruitment into the LP was detected before 4 days of exposure to DSS. At 48 and 72 hours after exposure to DSS, PMNs were largely absent from colonic tissues, and we were not able to visualize quantifiable levels of PMN migration.

To determine if the reduced motility of PMNs in the inflamed mucosa observed after induction of colitis by DSS was model specific, we examined PMN trafficking in another model of colitis induced by blocking IL-10 signaling and oral inoculation with *Helicobacter hepaticus* (20). *LysM^eGFP/+^* mice were gavaged with *H. hepaticus* for four consecutive days, and IL-10 receptors blocked by injecting anti-IL-10R Ab every 4 days over a 4-week period (Fig. S2A, Supplementary Material) (21). In contrast to a predilection for involvement of the distal colon in the DSS model, blockade of IL-10 following *H. hepaticus* infection induces robust inflammation mainly in the cecum and proximal colon (20) and (Fig. S2B, Supplementary Material). Blockade of IL-10R following *H. hepaticus* infection elicited robust PMN infiltration of the lamina propria compared to *H. hepaticus* alone as determined by histological analysis of colonic mucosa, quantification of lipocalin content in feces, and flow cytometry (Fig. S2B to D, Supplementary Material). Quantification of GFP^+^ cells in the lamina propria revealed that the majority of myeloid cells recruited following *H. hepaticus* gavage/IL-10R blockade were PMNs (Fig. S2E, Supplementary Material). Analysis of PMN migration into the proximal colon by IVM revealed low PMN motility, similar to results obtained following acute DSS-induced colitis (Fig. S2F and Movie S2, Supplementary Material). Comparable findings were further observed in IL10-deficient mice (*Il10^−^^/^^−^; LysM^eGFP/+^*) after gavage with *H. hepaticus*, demonstrating that results were not an artefact of repeated antibody injections (Fig. S2G and Movies S3 to S5, Supplementary Material).

### Biopsy-induced mucosal wounds result in massive PMN migration and swarming responses

**Fig. 3.**
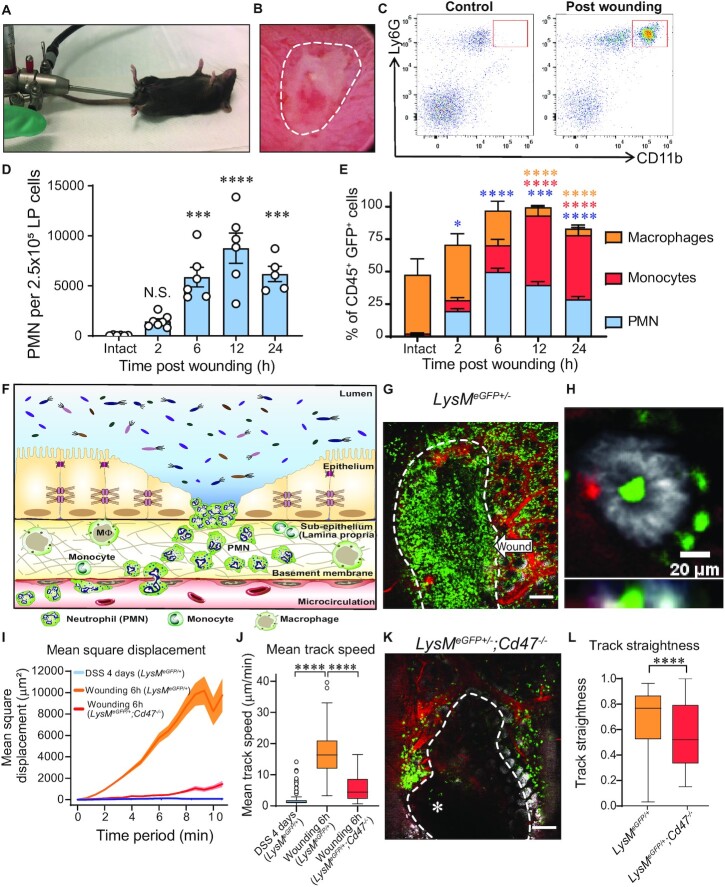
Acute mucosal wounds induce PMN swarming. (A and B) Rodent colonoscope and representative image of colonic biopsy-based wound. (C) Representative flow cytometry plot showing transmigrated PMNs (CD45^+^Ly6G^+^CD11b^high^) in wounded mucosa compared to intact tissue. (D and E) Quantification of wound-associated PMNs after biopsy-induced colonic injury (0 to 24 h). (D) Graph represents number of PMNs per 2.5 × 10^5^ cells of lamina propria cells. Data are means ± SEM of two independent experiments (*n* = 5 to 7 mice per group; ***P < 0.001; ^****^P < 0.0001 compared to Intact tissue by one-way ANOVA). (E) Represents percentage of wound associated GFP^+^ cells. Data are means ± SEM of two independent experiments (*n* = 5 to 7 mice per group; *P < 0.05; ***P < 0.001; ^****^P < 0.0001 compared to Intact tissue by two-way ANOVA. Asterisk colors represent compared groups matching figure keys). (F) Schematic overview of biopsy-wounding model: GFP^high^ PMNs swarming towards focalized, acute mucosal ulcer, characteristic of ulcerative colitis. (G) Representative image of GFP^high^ PMNs swarming towards a colonic biopsy wound (white-dashed line) (green = GFP, red = vasculature, white = EpCAM). White-dashed line limits the wounded area. Scale bar: 200 μm. (H) Representative image of PMNs interacting with intestinal epithelial cells (green = GFP, red = vasculature, white = EpCAM). Scale bar: 20 μm. (I and J) Comparison of PMN dynamics on DSS-treated and *LysM^eGFP/+^*vs. *Cd47^−^^/^^−^; LysM^eGFP/+^* wounded mice (tracks of at least 20 GFP^high^ PMN per mouse; *n* = 3 to 6 mice per group). (I) Mean square displacement and (J) Mean track speed. Data are represented as median (interquartile range). ^****^P < 0.0001 as determined by Wilcoxson–Mann–Whitney nonparametric test. (K) Representative images of PMN swarming towards a colonic mucosal wound on *Cd47^−^^/^^−^; LysM^eGFP/+^*mice (green: GFP, red: vasculature, white: EpCAM). White-dashed line limits wounded area. Asterisk points to migrating PMNs into wound. Scale bar: 100 μm. (L) Comparison of PMN track straightness on *LysM^eGFP/+^*vs. *Cd47^−^^/^^−^; LysM^eGFP/+^*wounded mice. Tracks of at least 20 GFP^high^ PMNs per mouse; *n* = 3 to 6 mice per group. Data are represented as median (interquartile range). ^****^P < 0.0001 as determined by Wilcoxson–Mann–Whitney nonparametric test.

We next investigated dynamics of PMN migration in an acute injury model that allows for direct assessment of immune cell recruitment following localized biopsy injury, under normal and colitic conditions. Using a miniaturized colonoscope, small biopsies were taken along the mucosa opposite to the mesenteric artery of the distal colon of *LysM^eGFP/+^* mice (Fig. 3A and B) (22). At indicated times between 6 and 24 h post-injury, wounds were excised, and immune cell influx quantified by flow cytometry. As detailed in Fig. 1C, PMNs that had migrated into colonic wounds in *LysM^eGFP/+^* mice were identified by Ly6G and high levels of CD11b surface expression (Fig. 3C). Analyses of kinetics of immune cell infiltration into wounds revealed that PMNs appeared as early as 2 h and numbers peaked 12 h post-wounding (Fig. 3D). Quantification of the relative abundance of PMNs, monocytes, and macrophages in excised wounds revealed at 6 h post-injury, GFP^high^ PMN represented the majority of infiltrated immune cells in colonic wounds (Fig. 3E). IVM imaging of PMN migration into the injured mucosa 6 h after biopsy-induced wounding revealed robust PMN swarming in response to colonic injury (Fig. 3F and G, and Movies S6 and S7, Supplementary Material). Importantly, in wound adjacent areas with intact epithelium, PMNs were observed interacting with and migrating in between intestinal epithelial cells (Fig. 3H, and Movie S8, Supplementary Material). Analyses of PMN trajectories revealed significantly increased mean square displacement and velocity in response to wounding in the distal colon compared to PMNs visualized in the inflamed lamina propria of mice exposed to DSS (Fig. 3I and J). To investigate whether injury-induced PMN swarming in the colon was microbiota-driven, we quantified wound associated PMNs in germ-free (GF) mice 6 h after biopsy induced wounding. As shown in the Supplementary Material Fig. S3, numbers of PMNs recruited to colonic wounds in GF mice were comparable to those observed in wounds from specific-pathogen-free (SPF) mice. These observations suggest that PMN colonic swarming observed after biopsy induced wounding is not stimulated by microbial invasion of the lamina propria that occurs after wounding.

We also investigated PMN migratory dynamics in mice deficient in CD47 (*Cd47^−^^/^^−^)* a known mediator of PMN TEpM and colonic wound repair (15, 22, 23). Consistent with recent reports, PMNs in *Cd47^−^^/^^−^; LysM^eGFP/+^* demonstrated significantly reduced migratory responses to biopsy wounding compared to *LysM^eGFP/+^* control mice as characterized by mean square displacement, speed, and directionality (Fig. 3I to L).

### Differences in PMN migratory dynamics remain unchanged after wounding during colitis

**Fig. 4.**
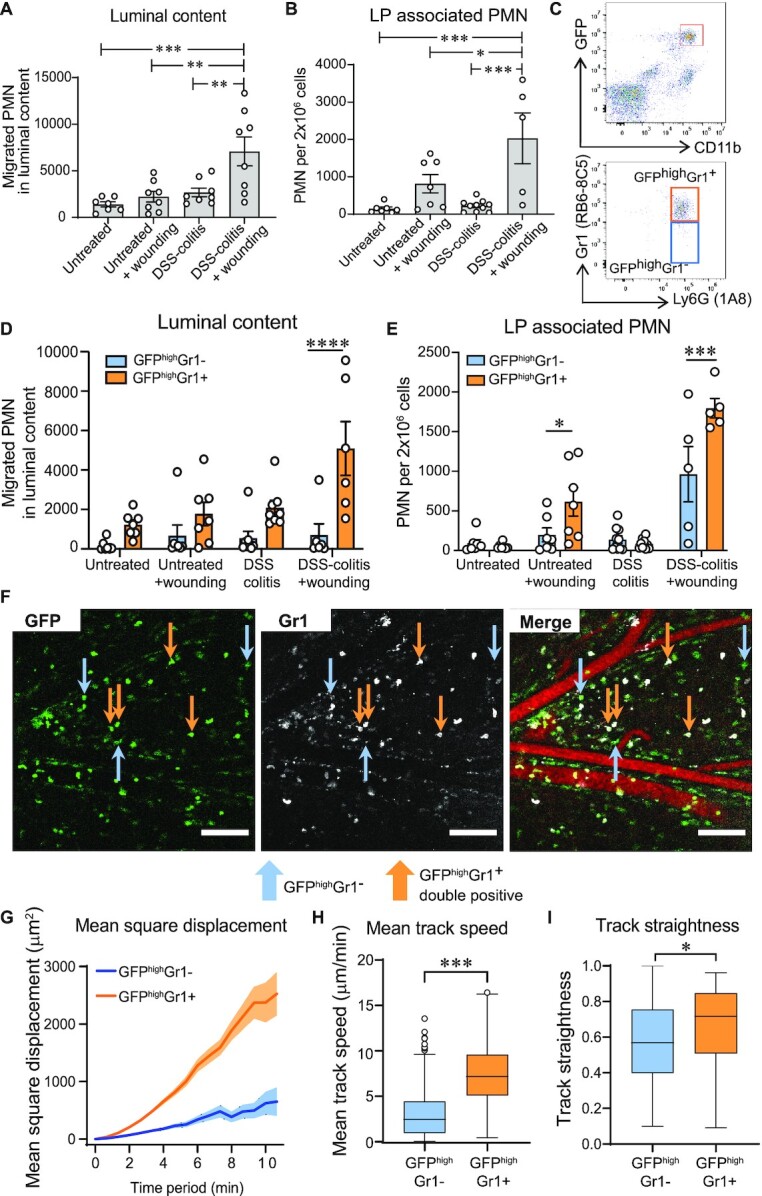
PMNs demonstrate different dynamics in response to mechanism and timing of mucosal injury. (A and B) Quantification of PMNs in the DCL model of mice submitted to different injury models as presented in Supplementary Table S1. (A) PMN TEpM into luminal content in response to LTB_4_. (B) Absolute numbers of PMNs infiltrating the lamina propria. Data is mean ± SEM of two independent experiments (*n* = 5 to 9 mice per group; *P < 0.05; **P < 0.01 ***P < 0.001 by one-way ANOVA). (C to E) Quantification of differentially labeled PMNs (GFP^high^Gr1^+^ and GFP^high^Gr1^−^) in the DCL model. (C) Gating strategy of the luminal content showing PMNs differentially stained with Gr1 after gating on CD45^+^GFP^+^CD11b^high^Ly6G^+^. (D) Gr1^+^ and Gr1^−^ PMNs transmigrated into the lumen of DCL in response to LTB_4_. (E) Absolute numbers of Gr1^+^ and Gr1^−^ PMNs infiltrating the lamina propria. Data are mean ± SEM of two independent experiments (*n* = 5 to 7 mice per group; *P < 0.05; ***P < 0.001; ^****^P < 0.0001 by two-way ANOVA). (F) Representative confocal-IVM images of DCL of mice subjected to DSS + wounding showing GFP (left) and Gr1staining (center). Merged channels (right) show two differentially stained PMNs: GFP^high^Gr1^−^ PMNs (blue arrows pointing at only green cells, as PMNs recruited into lamina propria in response to DSS), and double-labelling of GFP^high^Gr1^+^ PMNs (orange arrows pointing at green and white cells, as PMNs recruited from circulation in response to biopsy-wounding), as seen in the Supplemental Movie S9 (Green = GFP, white = Gr1, red = vasculature,). Scale bars: 100 μm. (G to I) Comparison of (G) Mean square displacement, (H) Mean track speed, and (I) Track straightness between GFP^high^Gr1^+^ and GFP^high^Gr1^−^ PMNs. Tracks of at least 20 PMNs per group. Data are represented as median (interquartile range) (*n* = 9 mice per group; *P < 0.05; ^****^P < 0.0001 as determined by Wilcoxson–Mann–Whitney nonparametric test).

Having identified differences in the PMN migratory response to foci of acute colonic injury compared to more diffuse injury resulting from acute DSS-induced colitis, we investigated effects of the inflammatory microenvironment on biopsy induced PMN swarming. To this end, PMN TEpM into the distal colon of *LysM^eGFP/+^*mice was compared in the groups listed in Supplementary Table S1. Biopsy-induced wounding during DSS-induced colitis significantly increased PMN TEpM into the intestinal lumen when compared to biopsy wounding or DSS-induced inflammation alone (Fig. 4A). These findings suggest that biopsy wounding provides an independent stimulus for recruitment of PMNs that had previously infiltrated the lamina propria in response to DSS-induced inflammation. Interestingly, in addition to increased luminal accumulation of PMNs, biopsy wounding of colitic mucosa elicited significantly more robust infiltration of PMNs into the lamina propria compared to biopsy injury or DSS colitis alone (Fig. 4B). We determined whether PMNs migrating into the lumen under the experimental conditions described in Fig. 4A were mobilized from the lamina propria or if they were recruited directly from the circulation. In addition to using CD11b surface expression as a distinct marker of transmigrating GFP^high^ Ly6G^+^ PMNs, we labeled intravascular PMNs at the time of wounding to further distinguish between circulating PMNs and lamina propria PMNs. Importantly, intravenous injection of anti-Gr1 mAb resulted in poor staining of mucosal PMNs (Fig. S4, Supplementary Material); however, robust staining of circulating PMNs with this antibody was observed (Fig. 4C and Fig. S4, Supplementary Material), providing a good measure of circulating versus lamina propria derived PMNs. Flow cytometry analysis revealed that the majority of PMNs migrating to the lumen of the distal colon following biopsy wounding in colitic mice were GFP^high^Gr1^+^ PMNs (Fig. 4D). These data indicate that biopsy wounding in colitic mice results in enhanced recruitment of circulating PMNs rather than recruitment of PMNs from the lamina propria (GFP^high^Gr1^−^).

Interestingly, there were significant numbers of PMNs newly recruited to the lamina propria in response to biopsy injury (GFP^high^Gr1^+^) compared to numbers of PMNs that had been recruited in response to DSS-induced inflammation alone (GFP^high^Gr1^−^) (Fig. 4E). IVM imaging of the submucosal space confirmed two distinct groups of PMNs, with GFP^high^Gr1^+^ double positive cells corresponding to PMNs migrating from the circulation into the lamina propria in response to biopsy-induced wounding (Fig. 4F; orange arrows), and GFP^high^Gr1^−^ cells representing PMNs recruited to the lamina propria in response to DSS (Fig. 4F; blue arrows). Tracking analyses revealed that GFP^high^Gr1^+^ PMNs displayed significantly increased motility as determined by greater displacement, increased track speeds, and enhanced directionality compared to GFP^high^Gr1^−^ PMNs (Fig. 4G to I and Movie S9, Supplementary Material).

### Distinct PMN dynamics correlate with differential surface expression of CXCR4 and senescence

**Fig. 5.**
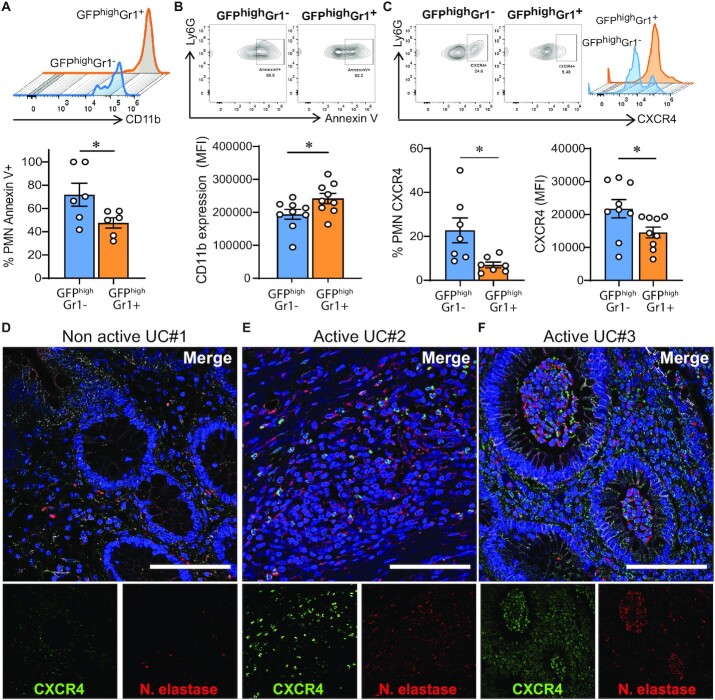
Different PMN dynamics correlates with different CXCR4 expression and apoptosis. Lamina propria PMNs were isolated 6 h post-wounding from mice subjected to DSS for 4 days. PMNs were identified by FACS as CD45^+^CD11b^+^Ly6G^+^. Two populations were distinguished based the presence or absence of Gr1^+^ staining. (A to C) Representative plots and quantification of expression of (A) CD11b, (B) apoptosis as determined by Annexin V binding, and (C) CXCR4 in GFP^high^Gr1^+^ and GFP^high^Gr1^−^ lamina propria PMNs from mice subjected to DSS + wounding. Data are mean ± SEM of two independent experiments (*n* = 5 to 7 mice per group; *P < 0.05; **P < 0.01 by two-tailed Student’s *t*-test). (D to F) Immunofluorescence staining of CXCR4 (green), Neutrophil elastase (red), and nuclei (blue) in colonic mucosa biopsies from patients with IBD. Representative confocal micrographs of (D) nonactive UC and (E and F) active UC. Scale bars: 100 μm.

Flow cytometric analyses of cell surface markers on PMNs localized in the lamina propria were performed to gain insights into observed differences in PMN migratory dynamics. Data revealed GFP^high^Gr1^+^ PMNs had significantly upregulated CD11b compared to GFP^high^Gr1^−^ PMNs (Fig. 5A). There was also a trend suggesting GFP^high^Gr1^+^ PMNs expressed less surface CD63 (a marker of degranulation) compared to GFP^high^Gr1^−^ PMNs, that fell just short of statistical significance (data not shown). Since PMNs responding to DSS-induced inflammation in the model may have been recruited to the lamina propria for several days, we investigated whether GFP^high^Gr1^−^ PMNs had phenotypes corresponding to senescence or cell death. Interestingly, increased Annexin V positivity was observed in GFP^high^Gr1^−^ compared to GFP^high^Gr1^+^ PMNs indicating development of apoptosis in these lamina propria leukocytes (Fig. 5B). Significantly increased expression of CXCR4 was also observed in the GFP^high^Gr1^−^ PMNs (Fig. 5C), consistent with recent reports demonstrating increased CXCR4 expression in aged PMNs (24, 25). While analyses of CXCR4 levels on circulating PMNs revealed no significant expression, there were increased levels of CXCR4 on lamina propria PMNs at day 4 after DSS, suggesting that upregulation of CXCR4 occurs following PMN extravasation and migration into the mucosa (Fig. S5A and B, Supplementary Material). It is well appreciated that CXCR4/CXCL12 receptor-ligand binding plays an important role in retaining PMNs within the bone marrow (26, 27). Therefore, we determined if the lower level of motility in GFP^high^Gr1^−^ PMNs is associated with increased CXCL12 expression. However, quantitative PCR analyses demonstrated no significant increase in CXCL12 expression in the lamina propria following exposure to DSS or after biopsy-wounding at any of the time points tested (Fig. S5C and D, Supplementary Material). Functional experiments were performed to determine whether blockade of CXCR4 signaling re-stimulates PMN colonic trafficking during DSS-induced inflammation. PMN TEpM into the distal colon was assessed after 4 days of DSS and treatment with the CXCR4 antagonist Plerixafor (AMD3100). While AMD3100 treatment resulted in mobilization of PMNs from bone marrow and increased circulating PMNs (Fig. S5E and F, Supplementary Material), intraperitoneal injection of AMD3100 2 h before intraluminal injection of LTB_4_ did not result in increased PMN TEpM into the gut lumen, suggesting that upregulation of CXCR4 is not acting as a retention signal in this intestinal model (Fig. S5G, Supplementary Material). Finally, we examined expression of CXCR4 on human PMNs in biopsies of inflamed colonic mucosa. As shown in Fig. 5D to F, increased expression of CXCR4 on PMN localized within the lamina propria and in crypt abscesses is observed in colonic mucosa from individuals with active UC compared to quiescent disease. These observations are consistent with upregulation of CXCR4 in mucosal PMNs during colitis in humans.

## Discussion

While precise regulation of PMN recruitment is essential for preventing infection and initiating tissue repair responses following inflammation or injury, dysregulated infiltration of PMNs into mucosal tissues is a hallmark of many chronic diseases, including IBD. Despite the important clinical consequences of PMN trafficking into epithelial lined organs, mechanisms directing PMN transepithelial migration remain incompletely understood. Since PMN activation and subsequent migratory behavior is greatly impacted by environmental cues, in-vivo systems represent promising tools to study PMN dynamics under real-life conditions. In this study, we describe an innovative in-vivo model that can be used to visualize PMN migratory behavior within the colonic mucosa during injury and repair using IVM. For imaging studies, we used *LysM^eGFP/+^* mice, an established mouse model previously used to visualize PMNs in vivo in other organs (28, 29). IVM assessment of PMN dynamics in the distal colon of *LysM^eGFP/+^* mice revealed specific migratory responses that differed depending on the nature of intestinal injury. Analyses revealed that PMNs recruited to the lamina propria during acute DSS-mediated injury failed to transmigrate to an additional chemotactic stimulus in the lumen of the distal colon. By contrast, PMNs recruited to the lamina propria following acute biopsy-induced injury demonstrated high motility and robust swarming towards biopsy sites. Previous studies have reported swarming responses upon injection of exogenous PMNs in skin injury models (30, 31). However, this study represents the first demonstration of extravascular swarming of PMNs in a (patho-)physiological environment in the gut and without additional transfer of exogenous PMNs.

In this work, we observed that the type of injury induced by different models resulted in distinct patterns of PMN motility. Whereas focal acute injury triggers a rapid infiltration of highly motile PMNs in a matter of hours, DSS induces progressive mucosal damage resulting in a gradual accumulation of PMNs that are relatively nonmotile after 4 days within the LP. In the DSS model, we found that PMNs failed to migrate in response to three different chemoattractants raising the possibility of an “exhausted” phenotype. It is also possible that PMNs have become desensitized in this model of DSS colitis and are unresponsive to further stimulation. It is well appreciated that effective PMN chemotaxis requires integration of multiple signals that rapidly regulates sensitivity to chemoattractants through expression of cell surface G-protein coupled receptors (GPCRs) (32), and/or through desensitization of GPCR signaling (33). GPCRs phosphorylation upon activation by agonists results in enhanced binding of beta-arrestin proteins that limits further interaction between agonists and GPCRs (34, 35). Such uncoupling or receptor desensitization represents a negative feedback loop to prevent overstimulation of PMNs (36).

Based on our results, we hypothesized that biopsy wounding following DSS-induced inflammation may serve as a secondary stimulus to reactivate PMNs present in the lamina propria. However, despite the application of a strong stimulus such as biopsy wounding, significant differences on PMN dynamics persisted as described above. Further analyses revealed that differences in PMN mobility observed following specific injuries corresponded to differences in surface expression of the leukocyte integrin CD11b, and the chemokine receptor CXCR4. The observation that CXCR4^+^ PMN in the lamina propria of mice treated with DSS were unable to mobilize in response to a secondary injury suggests the upregulation of CXCR4 in PMNs plays a functional role other than retaining PMNs in the tissue. To date, the effect of CXCR4 upregulation on PMN function outside of the bone marrow is not well understood. While previous work has shown that CXCR4 increases PMN retention in the lungs (37) and in wounded areas of a model of injury in zebrafish (38), administration of the CXCR4 antagonist AMD3100 did not increase PMN mobility in our study suggesting that CXCR4 does not act as a retention signal for PMNs in the gut. In support of organ specific functions for PMN CXCR4^+^, other studies have reported that CXCR4 upregulation in circulating PMNs is a sign of aging and facilitates migration back to the bone marrow for clearance (24, 25, 39, 40). Others have reported upregulation of CXCR4 on PMNs that have reverse transmigrated out of injured liver tissues, resulting in an increase of CXCR4^+^ and Annexin V^+^ injury-derived PMNs in the bone marrow (25). In contrast, others have used a model of endotoxemia to show that aged CXCR4^+^ circulating PMNs rapidly migrate to the site of inflammation rather than returning to bone marrow (41). There is controversy centered on whether distinct subpopulations of PMN exist or whether environmental cues determine phenotypic changes in PMNs over time (42–44). Therefore, we sought to determine if CXCR4 expression levels might help distinguish between discrete subsets of PMNs during colitic injury. Our data suggest that CXCR4 upregulation occurs after PMNs have migrated into the mucosa since we did not detect CXCR4 expression on circulating PMNs during DSS treatment. Such findings likely reflect PMN phenotypic plasticity in response to specific environmental stimuli. PMNs expressing CXCR4 exhibited lower motility and had increased rates of apoptosis, suggesting an advanced state of senescence and apoptosis. In support of this, Filippo and colleagues reported that upregulation of CXCR4 on human blood PMNs precedes apoptosis (45), and in-vitro aged human PMNs exhibit impaired migration and loss of proinflammatory functions such as reduced phagocytosis, and reduced fMLF-induced degranulation and respiratory burst (46). In contrast, in-vivo analyses in mice showed increased proinflammatory activity in aged PMNs while in the circulation (41, 47). However, our studies demonstrated upregulation of CXCR4 after PMNs were activated and had migrated into the mucosa. Further studies are clearly necessary to determine whether aged PMNs in tissue are more proinflammatory or less reactive than nonaged PMNs. Importantly, given the critical roles of PMNs and their clearance in host defense and wound healing (48, 49), increased senescence of lamina propria PMNs during colitis in combination with altered PMN function and impaired clearance could contribute to defective mucosal repair that is characteristic of IBD (50).

In conclusion, here, we demonstrate distinct PMN migratory dynamics in different models of acute intestinal inflammation/injury. We report that distinct patterns of PMN mobility correlate with surface expression of CXCR4. This new approach for examining dynamics of PMN migration in real time in the intestine will facilitate further studies to advance understanding of mechanisms regulating extravascular PMN motility and transepithelial migration in disease-relevant models. Furthermore, the ability to visualize dynamics of PMN trafficking in the distal colonic mucosa in real time will offer new approaches to validate therapeutic targets aimed at reducing aberrant PMN accumulation and tissue damage in the intestine.

## Methods

### Animals

Experimental studies were performed using WT (C57BL/6. Stock No: 000,664), *Il10^−^^/^^−^ H. hepaticus* free (B6.129P2-*Il10^tm1Cg^n*/J. Stock No: 002,251), and CD47-deficient (*Cd47^−^^/^^−^*) (B6.129S7-*Cd47^tm1Fpl^*/J. Stock No: 003,173) mice purchased from The Jackson Laboratory (Bar Harbor, ME, USA). *LysM^eGFP/eGFP^* mice (Lyz2^tm1.1Graf^) were a kind gift from Dr. Simon (University of California, Davis). Breeding colonies were established and *LysM^eGFP/+^, Il10^−^^/^^−^,LysM^eGFP/+^*, and *Cd47^−^^/^^−^;LysM^eGFP/+^* mice were housed at accredited University of Michigan animal resources facilities. Mice (10–14 weeks, male and female) were maintained under specific pathogen-free conditions with 12 h day/night cycle and access to food and water ad libitum. C57BL/6 GF and *Il10^−^^/^^−^;LysM^eGFP/+^* mice were maintained in *H. hepaticus* free and GF facilities, respectively. All experiments were approved and conducted in accordance with the guidelines of the Committee of Animal Research at the University of Michigan and the National Institutes of Health animal research guidelines as set forth in the Guide for the Care and Use of Laboratory Animals.

### Reagents

Ammonium–Chloride–Potassium (ACK) lysing buffer, EDTA 0.5 M, and HEPES 1 M were purchased from Lonza (Walkersville, MD, USA). Hanks’ Balanced Salt Solution with Ca^2+^ and Mg^2+^ (HBSS^+^), and Phosphate Buffered Saline without Ca^2+^ and Mg^2+^ (PBS^−^) were purchased from Corning Cellgro, Mediatech Inc. (Tewksbury, MA, USA). Rhodamine 70 kDa dextran, Hoechst 33,342 and CountBright counting beads were purchased from Invitrogen (Carlsbad, CA, USA). Fetal bovine serum (FBS) was obtained from Atlanta Biologicals (Oakwood, GA, USA). Leukotriene B_4_ (LTB_4_) was purchased from Cayman Chemical (Ann Arbor, MI, USA), N-Formylmethionyl-leucyl-phenylalanine (fMLF) and Plerixafor (AMD3100 octahydrochloride hydrate) were purchased from Millipore-Sigma (St. Louis, MO, USA). CXCL-1 was obtained from R&D Systems (Minneapolis, MN, USA). Recombinant mouse TNFα and IFNγ were purchased from Peprotech (Rocky Hill, NJ, USA). A complete list of antibodies clones and concentrations used in this work is provided as Supplementary Material (Supplementary Table S2).

### In-vivo wounding of colonic mucosa

Wounding of murine colonic mucosa was performed as described previously (51). Briefly, mice were anesthetized by intraperitoneal injection (i.p.) of a ketamine (80 mg/kg) and xylazine (5 mg/kg) solution. Biopsy-induced wounds in the distal colon were made along the mucosa opposite to the mesenteric artery using a miniaturized colonoscope system equipped with a biopsy-forceps coupled to a high-resolution camera (Colorview Veterinary Endoscope; Karl Stortz. El Segundo, CA, USA). Five to six biopsies were generated per mouse in order to acquire standardized levels of tissue injuries. For experiments combining biopsy wounding with DSS-induced colitis, immediately after wounding *LysM^eGFP/+^*mice were injected intravenously with 200 μl of PBS containing Alexa Fluor 647 conjugated antimouse Gr1 mAb (0.25 mg/mL; clone RB6-8C5. Invitrogen, #RM3021) to label circulating PMN. Subsequently, mucosal wounds were either used for IVM imaging or harvested at indicated time points for immune cell infiltration analysis.

### Dextran sulfate sodium (DSS)-induced colitis

Mice were provided with 2.5% w/v DSS (40 kDa, Affymetrix) in drinking water ad libitum for 2 to 6 days. Clinical disease assessment was obtained daily, with scores of 0 to 4 assigned for loss of body weight, stool consistency, and presence of blood in feces. Individual scores were added, and the average recorded as the disease activity index (DAI), with higher DAI values reflecting increasing severity of colitis (52).

### *H. hepaticus*-induced colitis

For *H. hepaticus*-induced colitis, mice were infected as described previously (53). Briefly, animals were inoculated by oral gavage with *H. hepaticus* (strain 51,449) for four consecutive days, followed by i.p. injection of 500 μg anti-IL-10R Ab (clone 1B1.3A. BioXcell. Lebanon, NH. #BE0050) every 4 days until day 28 post-inoculation. Similarly, *Il10^−^^/^^−^; LysM^eGFP/+^* mice were infected with *H. hepaticus* for four consecutive days but injection of anti-IL-10R Ab was not required. The inflammation in this model is mainly limited to the cecum and proximal colon (20). Disease activity was assessed by quantifying PMN gelatinase-associated lipocalin-2 in feces of infected animals by enzyme-linked immunosorbent assay (DuoSet Mouse Lipocalin-2/NGAL. R&D systems, #DY1857).

### Distal and proximal colon loop models

For in-vivo PMN recruitment studies, colitis was induced by 2.5% DSS or by *H. hepaticus* infection followed by IL-10R signaling blockade, and biopsy wounding of the colon as described above. The proximal colon loop (PCL) model was used to study inflammation in the *H. hepaticus* + anti-IL-10R mAb model of colitis as previously described (13). The DCL was specifically developed to study DSS-induced colitis and wound healing in the descending colon. Mice were anesthetized with isoflurane (Fluriso, VETONE. Boise, ID, USA) with a rodent vaporizer machine (EZ-7000; E-Z Systems Corporation. Palmer, PA, USA) using a heating pad to maintain body temperature. Abdominal disinfection was performed with alcohol swabs before a midline laparotomy was performed. Small bowel, caecum, and proximal colon were exteriorized without vascular disruption to provide access to the distal colon. Two mesocolonic incisions were made subsequently, one proximal to the superior mesenteric artery (SMA) and one distal to the inferior mesenteric artery (IMA). After opening the distal colon on both locations via small incisions, the lumen was gently flushed with warm HBSS^+^ to remove feces or potential blood clots caused by biopsy- or colitis-induced wounds and, to facilitate comparative analyses of luminal contents. Opened ends of the colon were subsequently ligated with nonabsorbable silk suture 3–0 (Braintree Scientific Inc. Braintree, MA, USA) to generate a 2 cm distal colon loop. To induce recruitment of PMN into the lumen of the distal colon, 200 μl of sterile HBSS^+^ alone or containing different chemoattractants (CXCL1 at 500 ng, fMLF at 10 μM, or LTB_4_ at 1 to 10 nM), were intraluminally injected using a 30-gauge needle (Becton Dickinson). All previously exteriorized organs were subsequently reinserted into the abdominal cavity and the abdomen itself was closed by wax-coated braided silk suture 3–0 (Sofsilk, #SS-694. Covidien. Minneapolis, MN, USA). After 60 min of incubation, the abdomen was reopened to excise the distal colon, and animals were euthanized by cervical dislocation. PMN that had migrated into the intestinal lumen of the distal colon loop, or PMN localized in the lamina propria were subsequently quantified by flow cytometry.

### Intravital microscopy

Confocal-IVM was performed following exteriorization of distal and proximal colon loops as described above. Animals were anesthetized, and before surgery a solution of 200 μl of HBSS^+^ containing Rhodamine 70 kDa dextran (1 mg/mL), Hoechst 33,342, and 10 μg of Alexa Fluor 647 conjugated anti-Gr1 mAb (clone RB6-8C5, Invitrogen, Carlsbad, CA, USA) was administered by tail vein injection to label blood vessels, nuclei and, circulating PMN. In some cases, Alexa Fluor 647 conjugated anti-EpCam mAb (clone G8.8. Biolegend. San Diego, CA, USA) was used to stain epithelial cells adjacent to wounds. Next, 50 μg Atropine (American Regent Inc, Shirley, NY, USA; #NDC0517-1010-25) was injected subcutaneously to reduce peristalsis, before mice were placed on a custom 3D-printed stage and transferred to a 37ºC heated chamber. Videos were acquired with a Leica SP5 upright confocal microscope (Leica Microsystems; Buffalo Grove, IL, USA) equipped with a 20 × HCX APO water-immersion lens and Leica Application Suite (LAS 2.7.3.9723). Excitation of all fluorescent probes used was achieved through a combination of a diode laser tuned at 405 nm, an argon gas laser tuned at 458 nm, and a tunable white light laser tuned at 561 and 633 nm. Emitted light was detected through a combination of Super-Sensitivity Hybrid Detector (HyD), SP mirrors set up at 415/460,498/545,571/637, and 657/778 nm, respectively, and Conventional Photo multiplier tubes (PMT) to generate four-color images. Videos were acquired by scanning 40 μm from the epithelial lining into the submucosa space every 40 s at 400 Mhz. Each plane represents an image of 512 × 512 (739.54 × 739.54 μm) in the *x* and *y* dimensions and nine sequential planes were acquired in the *z* dimension (5 μm per step) to form a *z* stack. Sequences of image stacks were transformed into 4D time-lapse movies using Fiji software (National Institute of Health, Bethesda, MD, USA). Cells were tracked by semiautomated tracking of cell motility in three dimensions (*x, y, t*) using the TrackMate plugin, and position data was imported into the online resource MotilityLab (hhtp://2ptrack.net) for parameter calculation, statistical analysis, and plotting using the R package CelltrackR explained previously in detail (54). Drift was corrected in Motility Lab using a stationary image feature or nonmotile cell as a reference track, and then tracks were pooled to calculated mean-squared displacement, cell speed and straightness. To minimize short and long track induced bias, track straightness was calculated using tracks between 6- and 15-time steps duration, Assessment of cell taxis (directionality) were based on individual image recording since the tissue wound and thus the direction of taxi may be oriented differently in each.

### PMN analysis in luminal content of colon loop models

PMN migration into the intestinal lumen was quantified as previously described (13, 15). Briefly, after collecting luminal contents and removing mucus through incubation with PBS^−^ containing 5 mM 1,4-dithiothreitol (DTT; Fisher BioReagents), cells were filtered through a 35 μm nylon mesh cell strainer (Falcon, #352,235). Cell suspension was centrifuged, washed, and transferred to a 96-well plate for subsequent immunostaining and flow cytometry analyses. Transmigrated luminal PMN were identified as CD45^+^CD11b^high^Ly6G^+^. Complete gating strategies are provided in Supplemental materials (Fig. S1, Supplementary Material).

### Harvesting of lamina propria cellular fraction by enzymatic digestion

Collection of the lamina propria fraction was performed as described previously (15). Briefly, distal colonic tissues were harvested, and mucus removed, followed by removal of intestinal epithelial cells, and mincing of remaining tissues for isolation of lamina propria enriched cell populations. For wound-associated cell analysis, four wounds per animal were removed, pooled together, and minced. Afterwards, lamina propria-enriched cell fractions were enzymatically digested with Liberase TM (2.5 mg/mL; Roche Diagnostics. Indianapolis, IN, USA), and DNase I (2 × 10^4^ Kuntz units/mL; MilliporeSigma. St Louis, MO, USA). Cells were counted, transferred to a 96-well plate, and stained for further analysis by flow cytometry. Complete gating strategies are provided as Supplemental materials (Fig. S4, Supplementary Material).

### Flow cytometry

Numbers of PMN and other myeloid cells were quantified within the different mucosal compartments, as follows. Cell suspensions were centrifuged, washed in PBS^−^ supplemented with 2% FBS, and incubated at 4ºC with mouse Fc Block for 15 min. Subsequently, a 30-min staining was performed at 4ºC in the dark with relevant antibody cocktails. For luminal content analysis, cells were stained with PerCP-CD45, PE-CD11b, and Alexa Fluor 647-Ly6G. PMN were identified as CD45^+^CD11b^high^Ly6G^+^. Myeloid cells isolated from LP were identified using PerCP-CD45, PE-CD11b, Brilliant Violet (BV)605-Ly6G, Alexa Flour 647-F4/80, PE-Cy7-Ly6-C, and BV785-CXCR4. Apoptosis was analyzed by probing cells with Pacific Blue conjugated Annexin V (1:20; #640,918, Biolegend) following the manufacturer’s instructions and analyzed by flow cytometry. Before acquisition, fluorescent counting beads (50 μl, #C36950. Invitrogen) were added according to the manufacturer’s recommendations, and samples were analyzed in a NovoCyte Flow Cytometer (ACEA Bioscience). Data analysis was performed using FlowJo v10 software (Tree Star, Ashland, OR, USA). A detailed description of antibodies used for flow cytometry analyses is provided in the Supplemental Materials.

### Immunofluorescence and histology

Colons were fixed with 10% formalin and embedded in paraffin. For hematoxylin and eosin stain (H&E) 4-μm sections were used to visualize inflammatory cell infiltration. Slides were scanned at the Slide Scanning Service/Digital Pathology Core, University of Michigan, with a Leica Biosystems-Aperio AT2 scanner equipped with a 0.75 NA Plan Apo 20× objective. A 40× scanning is achieved using a 2× magnification changer at 0.25 µm/pixel of resolution. Photomicrographs were captured using Aperio ImageScope 12 (Leica Biosystems).

Immunofluorescence staining of CXCR4 on human tissue, was performed on 6-μm paraffin-embedded sections slides of discarded resections from patients with ulcerative colitis, or negative colorectal screens obtained with approval from the University of Michigan IRB. After de-paraffinization, rehydration, and antigen retrieval with Antigen Unmasking Solution (1:100, Vector Laboratories, #H3301) using a decloaking chamber (1 min at 100°C, followed of 10 min at 90°C), tissue sections were permeabilized with 0.5% Triton X-100 in PBS for 10 min. Slides were incubated in blocking buffer (3% of Donkey serum in PBS + 0.05% Tween20) for 1 h at room temperature. Tissue was incubated with primary antibodies against CXCR4 (1:50, R&D systems, #MAB172) and neutrophil elastase (1:50; Abcam, Waltham, MA, USA, #ab68672) overnight at 4°C. Subsequently, secondary antibodies (1:500) were added at room temperature for 1 h and nuclei were counterstained with Hoechst 33,342 (1:2000, Invitrogen, #H3570). Slides were mounted with ProLong Gold Antifade reagent (Invitrogen, #P36934). Images were acquired with a Leica confocal microscope (Stellaris 5) equipped with a 20x/0.7NA and a HC PL APO CS2 63x/1.4NA, and Leica Application Suite (LAS 2.7.3.9723). FIJI software (NIH) was used for image processing.

### RNA isolation and real-time quantitative PCR

Total RNA was isolated from lamina propria cells using RNeasy Micro Plus kit (Hilden, Germany), followed by cDNA synthesis from 1 μg of total RNA with iScript^TM^ Reverse Transcription supermix (BioRad, Hercules, CA, USA). Semi-quantitative real-time PCR in the presence of SsoAdvance Universal SYBR Green Supermix (BioRad) was performed on the BioRad CFX Connect Real-time System using the following amplification program: 95°C for 2 min, 95°C for 5 s, 60°C for 30 s (× 40 cycles) followed by a melt curve program. Cq values were determined using a BioRad CFX Maestro 1.0 software. Relative mRNA expression was computed using the 2(-ΔΔCT) method and normalized to *Rps18* as reference gene. The following primers were used: *Cxcl12*: 25′-GGAGGATAGATGTGCTCTGGAAC-3′ (Forward) and 5′-AGTGAGGATGGAGA CCGTGGT G-3′ (Reverse); *Rps18*: 5′-ACTTTTGGGGCCTTCGTGTC-3′ (Forward) and 5′-GCCCAGAGACTCA TTTCTTCTTG-3′ (Reverse).

### Statistics

Statistical analyses were performed using GraphPad Prism software (GraphPad Software Inc.). Two-tailed Student’s *t*-test or Wilcoxon–Mann–Whitney *U* test were used to compare two samples normally distributed or nonparametric samples, respectively. One-way or two-way ANOVA analysis were used for multiple comparison. Bonferroni’s post-hoc test was used for nonparametric parameters. A *P* value less than 0.05 was considered statistically significant. Data are presented as means ± SEM whereby each dot represents one individual animal, and all results include at least two independent experiments.

### Study approval

All experimental procedures involving animals were conducted in accordance with the NIH guidelines and protocols approved by the University Committee on Use and Care of Animals at University of Michigan.

## Supplementary Material

pgac249_Supplemental_Files

## Data Availability

All data are available in the main text or the Supplemental Materials. All reagents used in this study were commercially available. All mouse strains used were available from the Jackson Laboratory unless otherwise indicated. *LysM^eGFP/eGFP^* mice (Lyz2^tm1.1Graf^) were available from Dr. Simon (University of California, Davis).

## References

[1] Mayadas TN , Cullere X, Lowell CA. 2014. The multifaceted functions of neutrophils. Annu Rev Pathol. 9:181–218. 24050624 10.1146/annurev-pathol-020712-164023 PMC4277181

[2] Peiseler M , Kubes P. 2019. More friend than foe: the emerging role of neutrophils in tissue repair. J Clin Invest. 129(7):2629–2639. 31205028 10.1172/JCI124616 PMC6597202

[3] Wang J. 2018. Neutrophils in tissue injury and repair. Cell Tissue Res. 371(3):531–539. 29383445 10.1007/s00441-017-2785-7 PMC5820392

[4] Kolaczkowska E , Kubes P. 2013. Neutrophil recruitment and function in health and inflammation. Nat Rev Immunol. 13(3):159–175. 23435331 10.1038/nri3399

[5] Fournier BM , Parkos CA. 2012. The role of neutrophils during intestinal inflammation. Mucosal Immunol. 5(4):354–366. 22491176 10.1038/mi.2012.24

[6] Parkos CA. 2016. Neutrophil–epithelial interactions: a double-edged sword. Am J Pathol. 186(6):1404–1416. 27083514 10.1016/j.ajpath.2016.02.001 PMC4901132

[7] Brazil JC , Parkos CA. 2016. Pathobiology of neutrophil–epithelial interactions. Immunol Rev. 273(1):94–111. 27558330 10.1111/imr.12446 PMC5000857

[8] Ley K , Laudanna C, Cybulsky MI, Nourshargh S. 2007. Getting to the site of inflammation: the leukocyte adhesion cascade updated. Nat Rev Immunol. 7(9):678–689. 17717539 10.1038/nri2156

[9] Leick M , Azcutia V, Newton G, Luscinskas FW. 2014. Leukocyte recruitment in inflammation: basic concepts and new mechanistic insights based on new models and microscopic imaging technologies. Cell Tissue Res. 355(3):647–656. 24562377 10.1007/s00441-014-1809-9 PMC3994997

[10] Kobayashi SD , Malachowa N, DeLeo FR. 2017. Influence of microbes on neutrophil life and death. Front Cell Infect Microbiol. 7:159. 28507953 10.3389/fcimb.2017.00159 PMC5410578

[11] Pillay J et al. 2010. In vivo labeling with ^2^H_2_O reveals a human neutrophil lifespan of 5.4 days. Blood. 116(4):625–627. 20410504 10.1182/blood-2010-01-259028

[12] Boerner K , Luissint AC, Parkos CA. 2021. Functional assessment of intestinal permeability and neutrophil transepithelial migration in mice using a standardized intestinal loop model. J Vis Exp. 11(168):e62093. 10.3791/62093 PMC11404721 33645571

[13] Flemming S , Luissint AC, Nusrat A, Parkos CA. 2018. Analysis of leukocyte transepithelial migration using an in vivo murine colonic loop model. JCI insight. 3(20):e99722. 30333307 10.1172/jci.insight.99722 PMC6237441

[14] Sumagin R , Robin AZ, Nusrat A, Parkos CA. 2014. Transmigrated neutrophils in the intestinal lumen engage ICAM-1 to regulate the epithelial barrier and neutrophil recruitment. Mucosal Immunol. 7(4):905–915. 24345805 10.1038/mi.2013.106 PMC4062590

[15] Azcutia V et al. 2021. Neutrophil expressed CD47 regulates CD11b/CD18-dependent neutrophil transepithelial migration in the intestine in vivo. Mucosal Immunol. 14(2):331–341. 32561828 10.1038/s41385-020-0316-4 PMC7749029

[16] Brazil JC et al. 2013. α3/4 Fucosyltransferase 3-dependent synthesis of Sialyl Lewis A on CD44 variant containing exon 6 mediates polymorphonuclear leukocyte detachment from intestinal epithelium during transepithelial migration. J Immunol. 191(9):4804–4817. 24068663 10.4049/jimmunol.1301307 PMC4047976

[17] Chtanova T et al. 2008. Dynamics of neutrophil migration in lymph nodes during infection. Immunity. 29(3):487–496. 18718768 10.1016/j.immuni.2008.07.012 PMC2569002

[18] Kreisel D et al. 2010. In vivo two-photon imaging reveals monocyte-dependent neutrophil extravasation during pulmonary inflammation. Proc Natl Acad Sci U S A. 107(42):18073–18078. 20923880 10.1073/pnas.1008737107 PMC2964224

[19] Nishi H et al. 2017. Neutrophil FcγRIIA promotes IgG-mediated glomerular neutrophil capture via Abl/Src kinases. J Clin Invest. 127(10):3810–3826. 28891817 10.1172/JCI94039 PMC5617671

[20] Kullberg MC et al. 1998. *Helicobacter hepaticus* triggers colitis in specific-pathogen-free interleukin-10 (IL-10)-deficient mice through an IL-12- and gamma interferon-dependent mechanism. Infect Immun. 66(11):5157–5166. 9784517 10.1128/iai.66.11.5157-5166.1998 PMC108643

[21] Kullberg MC et al. 2006. IL-23 plays a key role in *Helicobacter hepaticus*-induced T cell-dependent colitis. J Exp Med. 203(11):2485–2494. 17030948 10.1084/jem.20061082 PMC2118119

[22] Reed M et al. 2019. Epithelial CD47 is critical for mucosal repair in the murine intestine in vivo. Nat Commun. 10(1):5004. 31676794 10.1038/s41467-019-12968-y PMC6825175

[23] Parkos CA et al. 1996. CD47 mediates post-adhesive events required for neutrophil migration across polarized intestinal epithelia. J Cell Biol. 132(3):437–450. 8636220 10.1083/jcb.132.3.437 PMC2120714

[24] Martin C et al. 2003. Chemokines acting via CXCR2 and CXCR4 control the release of neutrophils from the bone marrow and their return following senescence. Immunity. 19(4):583–593. 14563322 10.1016/s1074-7613(03)00263-2

[25] Wang J et al. 2017. Visualizing the function and fate of neutrophils in sterile injury and repair. Science. 358(6359):111–116. 28983053 10.1126/science.aam9690

[26] Kim HK , De La Luz Sierra M, Williams CK, Gulino AV, Tosato G. 2006. G-CSF down-regulation of CXCR4 expression identified as a mechanism for mobilization of myeloid cells. Blood. 108(3):812–820. 16537807 10.1182/blood-2005-10-4162 PMC1895847

[27] Semerad CL et al. 2005. G-CSF potently inhibits osteoblast activity and CXCL12 mRNA expression in the bone marrow. Blood. 106(9):3020–3027. 16037394 10.1182/blood-2004-01-0272 PMC1895331

[28] Faust N , Varas F, Kelly LM, Heck S, Graf T. 2000. Insertion of enhanced green fluorescent protein into the lysozyme gene creates mice with green fluorescent granulocytes and macrophages. Blood. 96(2):719–726. 10887140

[29] Yam AO , Chtanova T. 2020. Imaging the neutrophil: intravital microscopy provides a dynamic view of neutrophil functions in host immunity. Cell Immunol. 350:103898. 30712753 10.1016/j.cellimm.2019.01.003

[30] Lammermann T et al. 2013. Neutrophil swarms require LTB4 and integrins at sites of cell death in vivo. Nature. 498(7454):371–375. 23708969 10.1038/nature12175 PMC3879961

[31] Ng LG et al. 2011. Visualizing the neutrophil response to sterile tissue injury in mouse dermis reveals a three-phase cascade of events. J Invest Dermatol. 131(10):2058–2068. 21697893 10.1038/jid.2011.179

[32] Borregaard N , Sorensen OE, Theilgaard-Monch K. 2007. Neutrophil granules: a library of innate immunity proteins. Trends Immunol. 28(8):340–345. 17627888 10.1016/j.it.2007.06.002

[33] Lammermann T , Kastenmuller W. 2019. Concepts of GPCR-controlled navigation in the immune system. Immunol Rev. 289(1):205–231. 30977203 10.1111/imr.12752 PMC6487968

[34] Pitcher JA , Freedman NJ, Lefkowitz RJ. 1998. G protein-coupled receptor kinases. Annu Rev Biochem. 67:653–692. 9759500 10.1146/annurev.biochem.67.1.653

[35] Steury MD , McCabe LR, Parameswaran N. 2017. G protein-coupled receptor kinases in the inflammatory response and signaling. Adv Immunol. 136:227–277. 28950947 10.1016/bs.ai.2017.05.003 PMC5730335

[36] Freedman NJ , Lefkowitz RJ. 1996. Desensitization of G protein-coupled receptors. Recent Prog Horm Res. 51:319–351.; discussion 52-3. 8701085

[37] Devi S et al. 2013. Neutrophil mobilization via plerixafor-mediated CXCR4 inhibition arises from lung demargination and blockade of neutrophil homing to the bone marrow. J Exp Med. 210(11):2321–2336. 24081949 10.1084/jem.20130056 PMC3804935

[38] Isles HM et al. 2019. The CXCL12/CXCR4 signaling axis retains neutrophils at inflammatory sites in zebrafish. Front Immunol. 10:1784. 31417560 10.3389/fimmu.2019.01784 PMC6684839

[39] Casanova-Acebes M et al. 2018. Neutrophils instruct homeostatic and pathological states in naive tissues. J Exp Med. 215(11):2778–2795. 30282719 10.1084/jem.20181468 PMC6219739

[40] de Oliveira S , Rosowski EE, Huttenlocher A. 2016. Neutrophil migration in infection and wound repair: going forward in reverse. Nat Rev Immunol. 16(6):378–391. 27231052 10.1038/nri.2016.49 PMC5367630

[41] Uhl B et al. 2016. Aged neutrophils contribute to the first line of defense in the acute inflammatory response. Blood. 128(19):2327–2337. 27609642 10.1182/blood-2016-05-718999 PMC5122310

[42] Deniset JF , Kubes P. 2018. Neutrophil heterogeneity: bona fide subsets or polarization states?. J Leukocyte Biol. 103(5):829–838. 29462505 10.1002/JLB.3RI0917-361R

[43] Rosales C. 2018. Neutrophil: a cell with many roles in inflammation or several cell types?. Front Physiol. 9:113. 29515456 10.3389/fphys.2018.00113 PMC5826082

[44] Grieshaber-Bouyer R et al. 2021. The neutrotime transcriptional signature defines a single continuum of neutrophils across biological compartments. Nat Commun. 12(1):2856. 34001893 10.1038/s41467-021-22973-9 PMC8129206

[45] De Filippo K , Rankin SM. 2018. CXCR4, the master regulator of neutrophil trafficking in homeostasis and disease. Eur J Clin Invest. 48 Suppl 2(Suppl 2):e12949. 29734477 10.1111/eci.12949 PMC6767022

[46] Whyte MK , Meagher LC, MacDermot J, Haslett C. 1993. Impairment of function in aging neutrophils is associated with apoptosis. J Immunol. 150(11):5124–5134. 8388425

[47] Zhang D et al. 2015. Neutrophil ageing is regulated by the microbiome. Nature. 525(7570):528–532. 26374999 10.1038/nature15367 PMC4712631

[48] Phillipson M , Kubes P. 2019. The healing power of neutrophils. Trends Immunol. 40(7):635–647. 31160208 10.1016/j.it.2019.05.001

[49] Brazil JC , Quiros M, Nusrat A, Parkos CA. 2019. Innate immune cell-epithelial crosstalk during wound repair. J Clin Invest. 129(8):2983–2993. 31329162 10.1172/JCI124618 PMC6668695

[50] Neurath MF , Travis SP. 2012. Mucosal healing in inflammatory bowel diseases: a systematic review. Gut. 61(11):1619–1635. 22842618 10.1136/gutjnl-2012-302830

[51] Quiros M et al. 2017. Macrophage-derived IL-10 mediates mucosal repair by epithelial WISP-1 signaling. J Clin Invest. 127(9):3510–3520. 28783045 10.1172/JCI90229 PMC5669557

[52] Krieglstein CF et al. 2002. Collagen-binding integrin alpha1beta1 regulates intestinal inflammation in experimental colitis. J Clin Invest. 110(12):1773–1782. 12488427 10.1172/JCI200215256 PMC151649

[53] Medina-Contreras O et al. 2016. Cutting edge: IL-36 receptor promotes resolution of intestinal damage. J Immunol. 196(1):34–38. 26590314 10.4049/jimmunol.1501312 PMC4684965

[54] Wortel IMN et al. 2021. CelltrackR: an R package for fast and flexible analysis of immune cell migration data. ImmunoInformatics. 1–2:100003. 10.1016/j.immuno.2021.100003 PMC10079262 37034276

