# Supplementary material for: Distinct stimulus-dependent neutrophil dynamics revealed by real-time imaging of intestinal mucosa after acute injury
Source: PNAS Nexus. 2022 Nov 4;1(5):pgac249. doi: 10.1093/pnasnexus/pgac249 (PMC9802210; doi:10.1093/pnasnexus/pgac249)
Supplement: pgac249_Supplemental_Files [file pgac249_supplemental_files.zip › PNASNEXUS-PNASNEXUS-2022-00768-s01.docx]

**Supplementary Material**

**Distinct stimulus-dependent neutrophil dynamics revealed by real-time imaging of intestinal mucosa after acute injury**

Veronica Azcutia,^a†^* Matthias Kelm,^a†‡^ Seonyoung Kim,^b^ Anny-Claude Luissint,^a^ Sven Flemming,^a‡^ Lisa Abernathy-Close,^c§^ Vincent B. Young,^c,d^ Asma Nusrat,^a^ Mark J. Miller,^b^ Charles A. Parkos.^a^*

* Address correspondence to:

Veronica Azcutia. University of Michigan Medical School, Department of Pathology.

109 Zina Pitcher Place, BSRB Room 4620, Ann Arbor, Michigan 48104, USA.

Phone: 734.936.1856; Email: [vazcucri@med.umich.edu](mailto:vazcucri@med.umich.edu);

Charles A Parkos, University of Michigan Medical School, Department of Pathology.

NCRC Building 35, Room 30-1537. 2800 Plymouth Road, Ann Arbor, Michigan 48109-2800, USA. Phone: 734.763.6384; Email: [cparkos@med.umich.edu](mailto:cparkos@med.umich.edu)

† V.A. and M.K. contributed equally to this work.

**This PDF file includes:**

Figures S1 to S5

Tables S1 to S2

Legends for Movies S1 to S9

**Other supplementary materials for this manuscript include the following:**

Movies S1 to S9

**
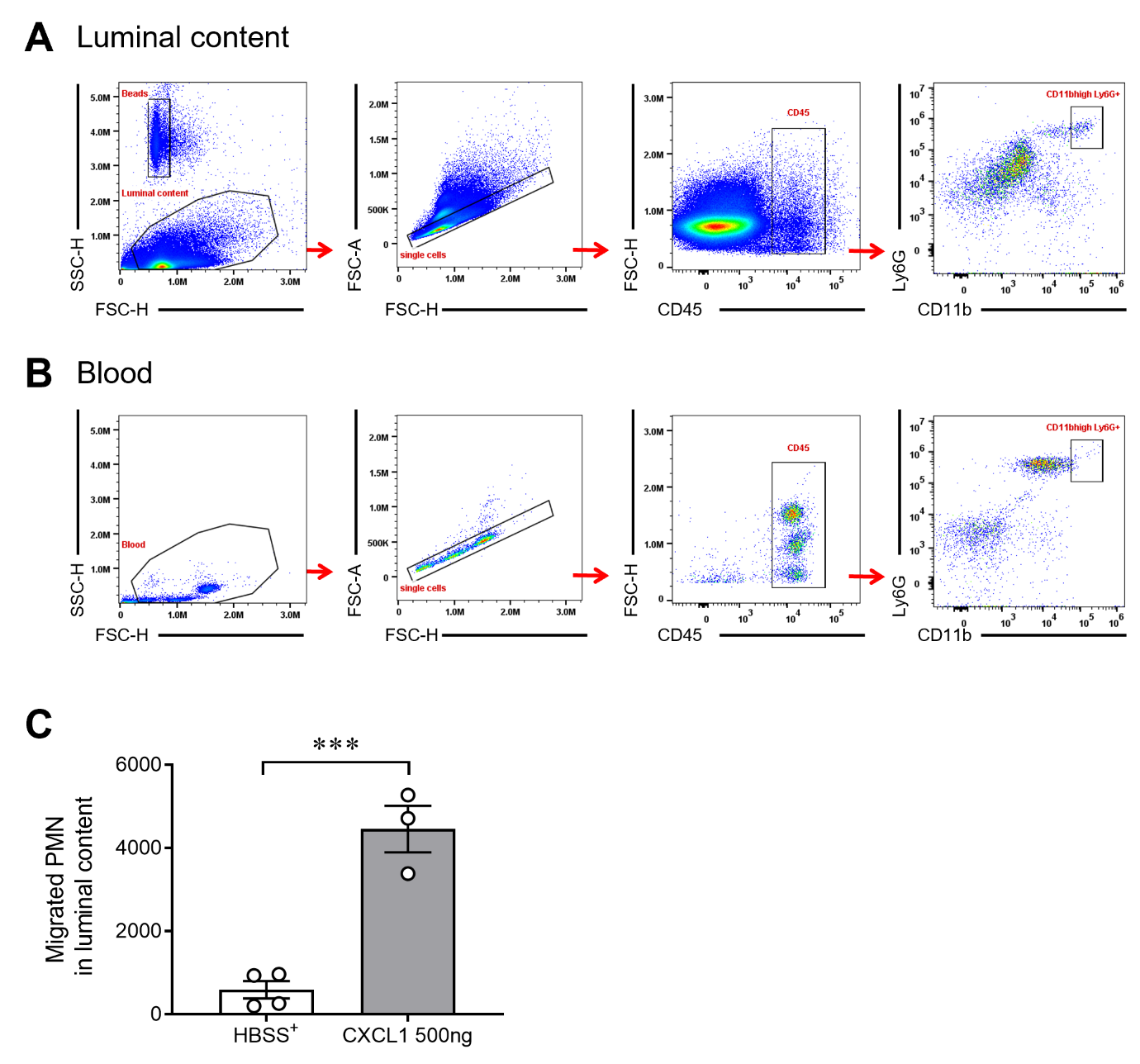
**

**Figure S1. Complete gating strategy for assessing migration of PMN into intestinal lumen.** (A) Gating strategy for identification of migrating PMN (CD45^+^CD11b^high^Ly6G^+^) in luminal content of DCL compared to (B) PMN from blood (CD45^+^CD11b^+^Ly6G^+^). (C) CXCL1 (500 ng) significantly increases PMN TEpM compared to control (HBSS^+^). Mice were injected i.p. with a cytokine cocktail containing IFNγ (100 ng) and TNFα (100 ng) 24 h before loop model. Graph represents quantification of migrated PMNs into luminal content by flow cytometry. Data are mean ± SEM (n=3-4 mice per group. *p<0.05 as determined by two-tailed Student’s t-test).

**
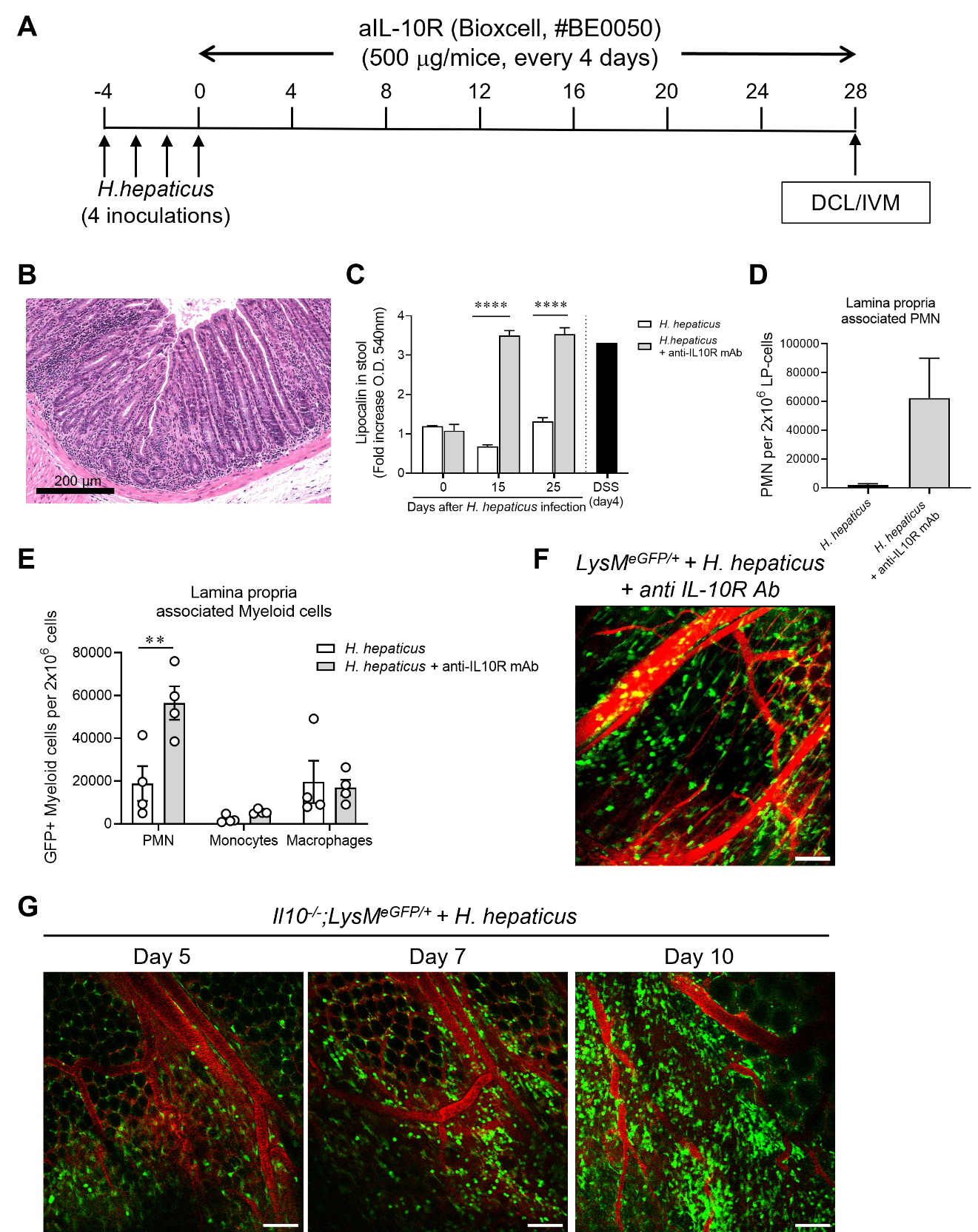
**

**Figure S2. PMN trafficking dynamics in the** ***H. hepaticus* + anti-IL-10R mAb colitis model.** (A) Scheme of the experimental design. (B) Representative H&E micrograph of the proximal colon demonstrating PMN infiltration into the colonic mucosa of mice infected with *H. hepaticus* + anti-IL-10R mAb at day 28. Scale bar: 200 μm. (C) Levels of Lipocalin-2/NGAL measured in feces at different days after infection with *H*. *hepaticus* alone (white bars) or *H. hepaticus* + anti-IL-10R mAb (gray bars). Mice exposed to DSS for four days (black bar) served as a positive control. Data are means ± SEM (n= 4 mice per group. **** p<0.0001 by ANOVA). (D) Quantification of PMN infiltrating lamina propria of the proximal colon loop (PCL) at day 28 post-infection. Data are means ± SEM (n = 2 independent experiments). (E) Quantification of GFP+ myeloid cells accumulated in lamina propria at day 28 post-infection. Data are means ± SEM of 2 independent experiments (n=2-4 mice per group. **p<0.01 by two-way ANOVA). (F) Representative image of confocal-IVM movie from *LysM^eGFP/+^* mice at day 28 post-infection with *H. hepaticus* and injected with anti-IL-10R mAb. (G) Representative images of confocal-IVM movies from *Il10^-/-^*;*LysM^eGFP/+^* mice infected with *H. hepaticus* at different days post-infection. Green (GFP), red (vasculature). Scale bars: 100 μm.

**
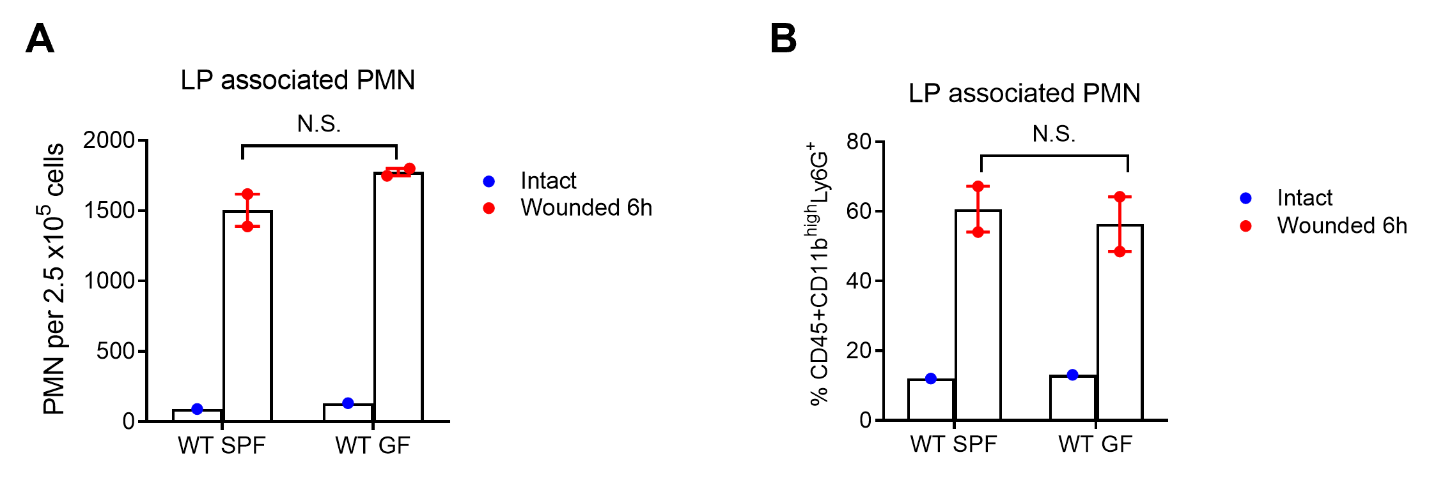
**

**Figure S3. No difference in PMN recruitment into germ-free mucosal wounds compared to SPF mice.** Quantification of wound-associated PMNs (CD45^+^CD11b^high^Ly6G^+^) 6 h after biopsy-wounding on SFP and GF mice. (A) Total number and (B) Percentage of lamina propria PMNs (n=2 mice per group. N.S. differences between SPF and GF mice by two-way ANOVA).

**
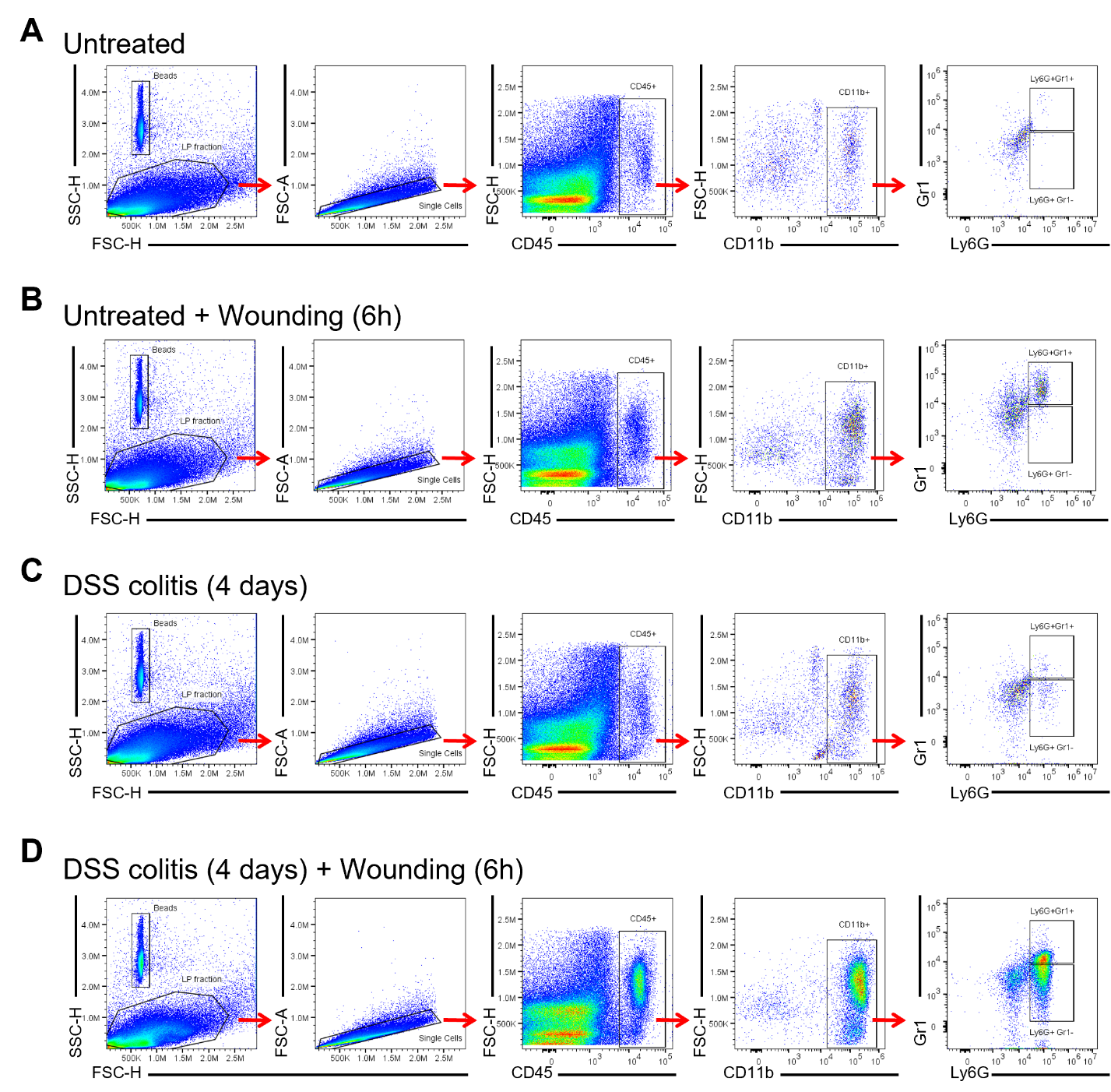
**

**Figure S4. Complete gating strategy for identification of PMN pools in relevant inflammatory models.** Detailed gating strategy for PMN differentiation after inflammatory stimulus described in Table S1.

**
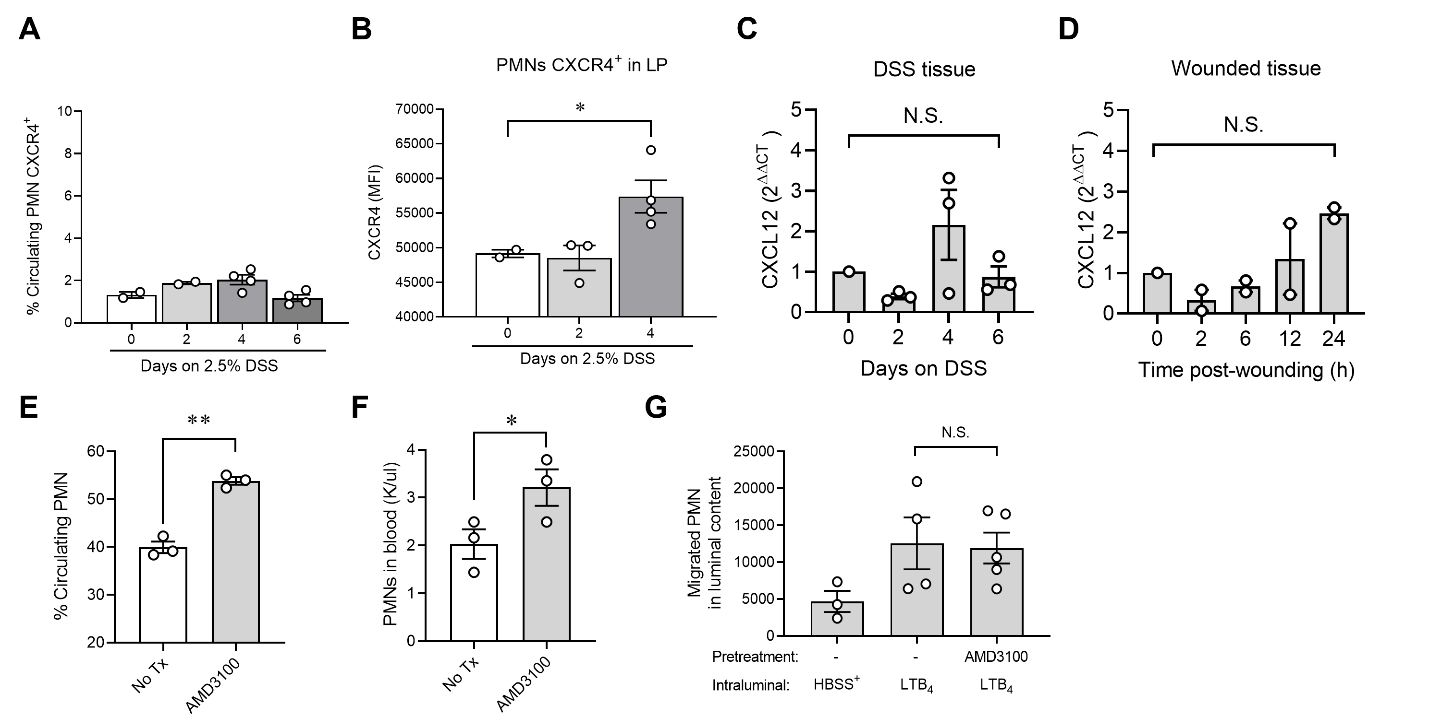
**

**Figure S5. CXCR4/CXCL12 axis is not responsible of PMN retention in lamina propria.**  (A) Percentage of peripheral blood PMN expressing CXCR4 in mice subjected to DSS (0-6 days) and (B) CXCR4 levels on PMN isolated from LP after DSS exposure (0-4 days) Data are means ± SEM (n=2-4 mice per group. *p=0.05 by ANOVA). (C and D) Quantification of CXCL12 mRNA by qPCR on DSS-inflamed or biopsy-wounded mucosal tissue. Data are means ± SEM (n=2-3 mice per group. N.S. differences between groups by ANOVA). (E and F) Percentage and numbers of circulating PMN in mice 2h after i.p. injection of the CXCR4 antagonist AMD3100 (5 mg/kg). Data are means ± SEM (n=3 mice per group. *p<0.05; **p<0.01 by two-tailed Student’s t-test). (G) Absolute numbers of PMN migrating to luminal content in the DCL model in response to luminal application of LTB_4_. Some mice received an i.p. injection of AMD3100 (5 mg/kg) 2 h before DCL assay. Data are means ± SEM (n=3-5 mice per group. N.S. differences between groups by ANOVA).


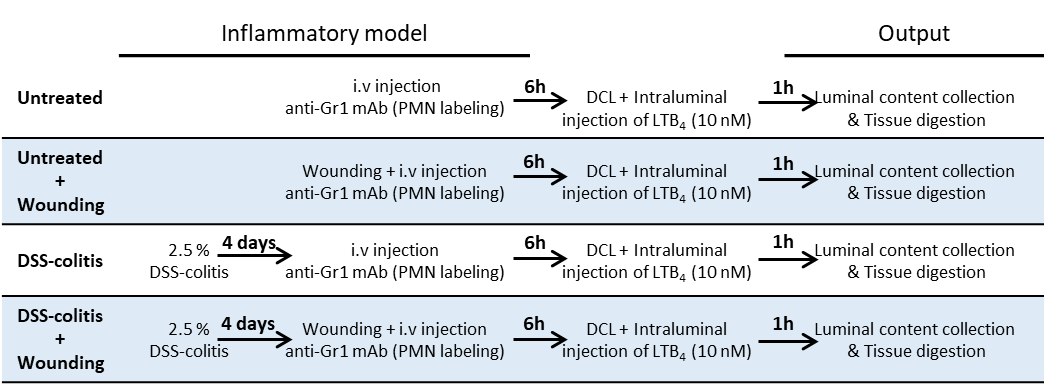


**Table S1. Scheme of the different groups subjected to different intestinal inflammatory models.** Group 1, untreated mice. Group 2, biopsy-wounded mice for 6 h. Group 3, mice treated with 2.5% of DSS for 4 days. Group 4, mice treated with 2.5% of DSS for 4 days followed by biopsy-wounding for 6 h. At the time corresponding to biopsy wounding on group 2 and 4, all groups of mice were i.v. injected with 50 μg of Alexa Fluor-647 anti-Gr1 mAb to label circulating PMN. DCL was performed in all groups and PMN TEpM was stimulated by instilling a solution of HBSS^+^ containing 10 nM of LTB_4_ for 1h. Subsequently, PMN migrating into luminal content and lamina propria were quantified by flow cytometry.


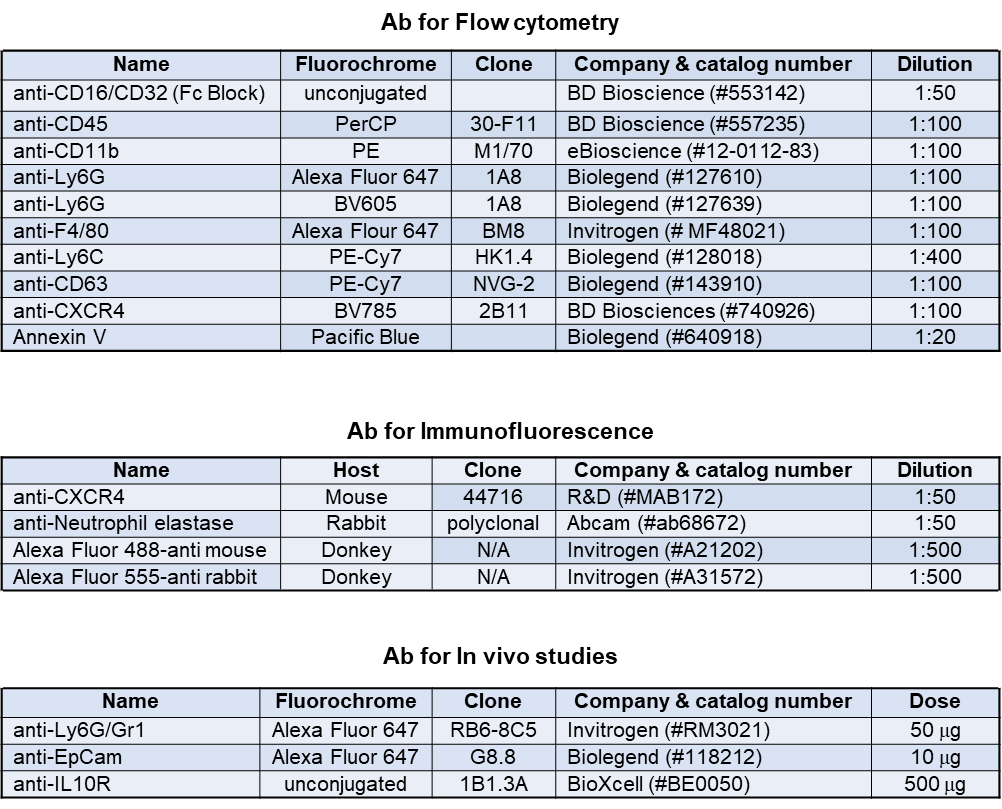


**Table S2. List of antibodies used in this study.**

**Movie S1: PMN migratory dynamics during DSS-induced colitis.** IVM revealed low PMN motility in the distal colon in response to intraluminal application of LTB_4_ (10nM) in acute DSS-induced colitis. Green (GFP), red (vasculature). Scale bar: 200 μm.

**Movie S2: PMN migratory dynamics during *H. hepaticus* + IL-10R mAb -induced colitis.** Analysis of PMN migration into the proximal colon by IVM revealed low PMN motility in a model of colitis induced by *H. hepaticus* + IL10 signaling blockade followed by intraluminal injection of LTB_4_ (10nM), similar to results obtained following acute DSS-induced colitis. Green (GFP), red (vasculature). Scale bar: 200 μm.

**Movie S3: PMN migratory dynamics in *Il10^-/-^* mice 5 days after *H. hepaticus*-infection.** Video shows the proximal colon loop of *Il10^-/-^*;*LysM^eGFP/+^* mice in a model of colitis induced by *H. hepaticus*. Images reveal low numbers of PMN infiltrated in the LP on day 5 post-infection, and mucosal PMNs demonstrate low motility in response of intraluminal injection of LTB_4_ (10nM). Green (GFP), red (vasculature). Scale bar: 100 μm.

**Movie S4: PMN migratory dynamics in *Il10^-/-^* mice 7 days after *H. hepaticus*-infection.** Imaging of the proximal colon loop of *Il10^-/-^*;*LysM^eGFP/+^* mice in a model of colitis induced by *H. hepaticus*. On day 7 post-infection there are increased numbers of PMN infiltrating the LP of the PCL. Despite the increase in PMNs, there is low motility in response to intraluminal injection of LTB_4_ (10nM). Green (GFP), red (vasculature). Scale bar: 100 μm.

**Movie S5: PMN migratory dynamics in *Il10^-/-^* mice 10 days after *H. hepaticus*-infection.** IVM of *Il10^-/-^*;*LysM^eGFP/+^* mice in a model of colitis induced by *H. hepaticus* on day 10 post-infection reveals robust inflammation of the PCL characterized by high numbers of PMNs that remain in a low motile state upon intraluminal injection of LTB_4_ (10nM). Green (GFP), red (vasculature). Scale bar: 100 μm.

**Movie S6: PMN migration across the intestinal mucosa: transendothelial, interstitial, and transepithelial migration.** IVM allows imaging GFP^high^ PMNs transmigrating across different barriers. PMNs, in response to biopsy-induced wounding, leave the circulation crossing the vascular linings (transendothelial migration or TEM), traverse the submucosa space (interstitial migration), and interact with intestinal epithelial cells to traverse the mucosal barrier towards the luminal space (transepithelial migration or TEpM). Green (GFP), red (vasculature), white (EpCAM). Scale bar: 100 μm.

**Movie S7: PMN swarming in response to biopsy-wounding.** IVM imaging of GFP^high^ PMNs swarming into colonic injury at 6 h after biopsy-induced wounding. White line indicates the mucosal wound contour. Green (GFP), red (vasculature), white (EpCAM). Scale bar: 100 μm.

**Movie S8: PMN interaction with intestinal epithelial cells.** IVM of the distal colon loop is a useful tool to visualize PMN-Intestinal Epithelial Cell (IEC) interactions. In adjacent areas of mucosal wounds, GFP^high^ PMNs were observed interacting with and migrating in between intact intestinal epithelial cells. Green (GFP), red (vasculature), white (EpCAM). Scale bar: 20 μm.

**Movie S9: Distinct stimulus-dependent PMN dynamics following biopsy-wounding on DSS colitic mucosa.** IVM of distal colon loop 6h after biopsy-wounding the colitic mucosa showed enhanced GFP^high^ PMN infiltration of the lamina propria. Differential staining of circulating PMNs with anti-Gr1 Ab at the time of wounding revealed that the majority of PMNs recruited directly from the circulation in response to mucosal wounding were highly motile (GFP^high^Gr1^+^; white and green cells), whereas GFP^high^Gr1^-^ PMNs that infiltrated the mucosa in response to DSS before wounding represent an immotile pool of PMNs (GFP^high^Gr1^-^ only-green cells). Green (GFP), white (Gr1), red (vasculature). Scale bar: 100 μm.
